# Suspected Sexual Transmission of Dermatophilosis among Men Who Have Sex with Men, Barcelona, Spain, 2025–2026

**DOI:** 10.3201/eid3206.260476

**Published:** 2026-06

**Authors:** Vicente Descalzo, Albert Moreno-Mingorance, Patricia Álvarez-López, Paula Salmerón, Jorge N. García-Pérez, Ferran P. Pericás-Cladera, Jordi Arcarons, Guillem Puigsech-Boixeda, Ilaria Furlan, Ester del Barrio-Tofiño, Sol M. San José, Nieves Larrosa, Vicenç Falcó, Mayli Lung, Maider Arando, Juan José González-López

**Affiliations:** Vall d’Hebron University Hospital, Barcelona, Spain (V. Descalzo, P. Álvarez-López, P. Salmerón, J.N. García-Pérez, F.P. Pericás-Cladera, J. Arcarons, G. Puigsech-Boixeda, I. Furlan, E. del Barrio-Tofiño, S.M. San José, N. Larrosa, V. Falcó, M. Lung, M. Arando, J.J. González-López); Universitat Autònoma de Barcelona, Barcelona (V. Descalzo, N. Larrosa, V. Falcó, M. Lung, M. Arando, J.J. González-López); Vall d’Hebron Research Institute, Barcelona (A. Moreno-Mingorance, P. Salmerón, F.P. Pericás-Cladera, G. Puigsech-Boixeda, E. del Barrio-Tofiño, S.M. San José, N. Larrosa, V. Falcó, M. Lung, J.J. González-López); CIBER of Infectious Diseases (CIBERINFEC), Madrid, Spain (A. Moreno-Mingorance, G. Puigsech-Boixeda, N. Larrosa, M. Lung, J.J. González-López)

**Keywords:** dermatophilosis, bacteria, zoonoses, Dermatophilus congolensis, sexually transmitted infections, sexual transmission, sexual and gender minorities, men who have sex with men, skin diseases, Spain

## Abstract

Dermatophilosis is considered a zoonotic infection. We report 9 cases among men who have sex with men in Barcelona, Spain, during December 2025–March 2026. Whole-genome sequencing revealed highly related isolates forming a distinct lineage within the genus *Dermatophilus*. Epidemiologic and clinical features of the cases support human-to-human transmission via sexual contact.

Dermatophilosis is a skin infection caused by *Dermatophilus congolensis*, a gram-positive actimoycete that primarily affects animals in tropical and subtropical regions (*1*). Human infections are rare and typically associated with zoonotic exposure to livestock or wildlife; manifestations include pitted keratolysis, folliculitis, and pustules (*2*–*6*). Human-to-human transmission has not been documented. Here, we report 9 cases of dermatophilosis diagnosed in Barcelona, Spain, and assess the possibility of autochthonous human-to-human transmission via sexual contact.

## The Study

The cases involved 2 patients who attended primary healthcare centers in December 2025 and March 2026 and 7 patients who attended a reference clinic for sexually transmitted infections (STIs) during January–March 2026 (Table). All patients were cisgender men who reported having sex with men (MSM). The median age was 47 years (interquartile range 30.5–57.5 years). Four patients were HIV-positive and 3 were receiving HIV preexposure prophylaxis therapy. Four patients had concomitant STIs, and 3 reported engaging in chemsex (drug use in a sexual context).

**Table:** Epidemiologic and clinical characteristics of dermatophilosis cases in men who have sex with men, Barcelona, Spain, December 2025–March 2026*

Pt no.	Age, y	HIV	Travel in previous month	Chemsex	Visited sex venue in week before symptom onset	Sexual partner with symptoms	STI co-infection	Symptom duration, d	Infection site	Lesion types	Treatment
1	61	N	London, UK	N	Y, sauna	Y	Early latent syphilis	15	Thorax	Vesicles, Pustules	Benzathine penicillin
2	27	N, on PrEP	Toulouse, France	N	Y, sauna	Y	N	5	Beard, penis, scrotum, thigh	Papules, vesicles, pustules	Mupirocin, doxycycline
3	47	Y, on ART; CD4 495 cells/mm^3^	N	Y, CM	Y, sex club	N	Early latent syphilis	10	Buttock, scrotum, groin	Scaly papules	Cefadroxil
4	49	N, on PrEP	Vienna, Austria; Budapest, Hungary	N	Y, sauna	Y, pt. no. 5	N	6	Beard, penis, groin	Pustules, nodules	Cefadroxil
5	54	N, on PrEP	Vienna, Austria; Budapest, Hungary	N	Y, sauna	Y, pt. no. 4	N	3	Thigh, armpit	Vesicles, pustules	Cefadroxil
6	32	N	No	N	Y, sauna	N	NG, LGV, HSV-2	45	Thigh	Nodules, scaly lesions	Ceftriaxone, doxycycline
7	66	Y, on ART; CD4 217 cells/mm^3^	N	Y, CM, GHB, 4-MMC	Y, sauna	N	No	15	Beard	Scaly papules, scabs	Cefadroxil
8	29	Y, on ART; CD4 860 cells/mm^3^	N	N	Y, sauna	N	NG, CT	4	Scrotum	Pustules, scabs	Cefadroxil
9	37	Y, on ART; CD4 475 cells/mm^3^	N	Y, CM, GHB	Y, sauna	N	N	5	Perianal, groin, thigh	Scaly papules, pustules, scabs	Cloxacillin

*4-MMC, mephedrone; ART, antiretroviral therapy; chemsex, drug use in a sexual context; CM, methamphetamine; CT, *Chlamydia trachomatis*; GHB, gamma-hydroxybutyric acid; HSV-2, herpes simplex virus type 2; LGV, lymphogranuloma venereum; NG, *Neisseria gonorrhoeae*; PrEP: preexposure prophylaxis; STI, sexually transmitted infection; pt., patient.

None of the patients reported contact with livestock or wildlife, and none had traveled to tropical regions in the month before symptom onset. Four patients reported recent travel to other cities in Europe where they engaged in sexual activity. All patients reported visiting venues for sexual encounters within the week preceding symptom onset, including 8 who had visited a sauna. Two patients were regular sexual partners, and 2 others reported partners with similar symptoms who were treated in other centers without microbiological confirmation.

**Figure 1:**
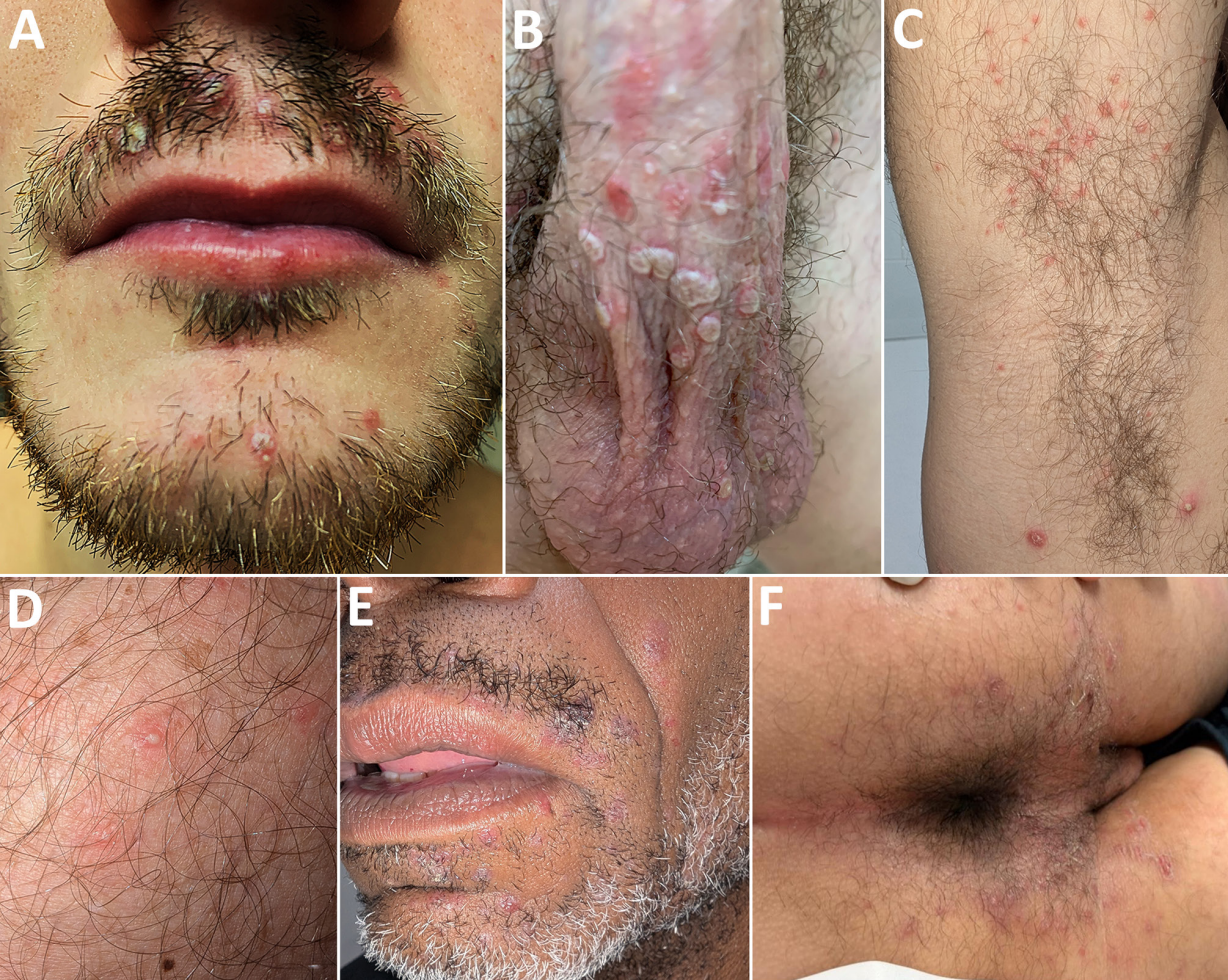
Dermatophilosis lesions in patients in cluster of suspected sexual transmission among men who have sex with men, Barcelona, Spain, December 2025–March 2026. A) Papule-pustules on the beard area; B) papulovesicular lesions on the penis and scrotum; C) pustular rash in the armpit; D) vesicle-pustules on the thigh; E) erythematous scaling papules on the beard area; F) erythematous scaling papules in the perianal region.

Median symptom duration at consultation was 6 days (interquartile range 4.5–15 days). Lesions manifested as an itchy folliculitis-like rash characterized by papules, vesicles, pustules, scabs, nodules, or scaly lesions (Figure 1). Multiple anatomic sites were affected, most commonly the genitals, thighs, groin, and beard area.

Five patients were treated with cefadroxil (500 mg 2×/d for 7 d) resulting in complete resolution of lesions in all but 1 case, who had a residual nodular lesion. One patient was successfully treated with cloxacillin (500 mg/6 h for 7 d). Two patients showed cleared lesions after treatment for concomitant STIs (benzathine penicillin G for syphilis and ceftriaxone and doxycycline for gonorrhea and lymphogranuloma venereum). One patient initially received topical mupirocin with partial response but achieved complete resolution after doxycycline therapy (100 mg 2×/d for 7 d). None of the patients required hospitalization or experienced any complications. As of April 2026, no recurrences had been observed.

Skin lesion samples cultured on Columbia 5% sheep blood agar produced small yellowish β-hemolytic colonies. Gram staining revealed gram-positive coccoid forms occasionally arranged in irregular filaments with transverse septa. Identification by matrix-assisted laser desorption/ionization time-of-flight mass spectrometry confirmed *Dermatophilus congolensis*.

All isolates showed identical antimicrobial susceptibility profiles and were susceptible to amoxicillin/clavulanate (MIC <2/1 mg/L), ceftriaxone (MIC <4 mg/L), cefepime (MIC <1 mg/L), imipenem (MIC <2 mg/L), linezolid (MIC 1 mg/L), amikacin (MIC 4 mg/L), moxifloxacin (MIC <0.25 mg/L), doxycycline (MIC <0.12 mg/L), minocycline (MIC <1 mg/L), clarithromycin (MIC <0.06 mg/L), and trimethoprim/sulfamethoxazole (MIC <0.25/4.75 mg/L). All isolates were resistant to tobramycin (MIC 16 mg/L) and showed intermediate susceptibility to ciprofloxacin (MIC 2 mg/L). We interpreted susceptibility on the basis of CLSI 2023 breakpoints for other aerobic actinomycetes (*7*) because no organism-specific breakpoints are available for *D. congolensis*. MIC for mupirocin was >1,024 mg/L for all isolates; no clinical breakpoints are available.

**Figure 2:**
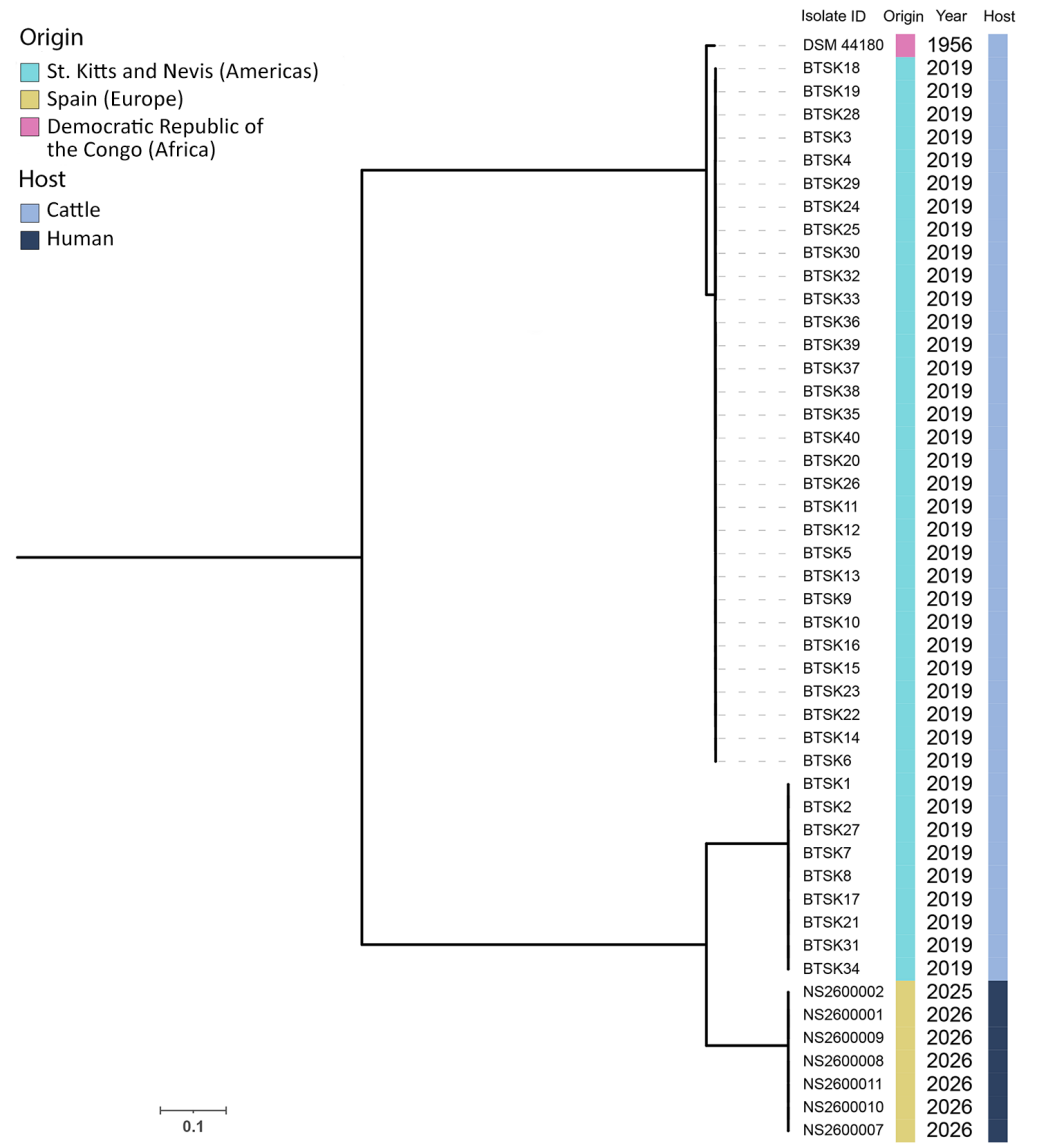
Phylogenetic analysis of *Dermatophilus* isolates in cluster of suspected sexual transmission of dermatophilosis among men who have sex with men, Barcelona, Spain, December 2025–March 2026. Maximum-likelihood phylogenetic tree shows *Dermatophilus* isolates from patient nos. 1–7 obtained in this study (bottom of tree) compared with 40 genomes from cattle in Saint Kitts and Nevis (*8*), and reference strain *D. congolensis* DSM 44180 (all from the National Center for Biotechnology Information Sequence Read Archive, https://www.ncbi.nlm.nih.gov/sra). Scale bar indicates nucleotide substitutions per site.

We performed whole-genome sequencing (WGS) on isolates from patients 1–7. WGS demonstrated extremely close genetic relatedness among isolates; pairwise single-nucleotide polymorphism (SNP) distances were 0–4 SNPs. We also performed phylogenetic comparisons between the isolates from this study and publicly available *D. congolensis* genomes, including 40 genomes from cattle in Saint Kitts and Nevis (*8*) and reference strain *D. congolensis* DSM 44180 from the Democratic Republic of the Congo, all from the National Center for Biotechnology Information Sequence Read Archive (https://www.ncbi.nlm.nih.gov/sra). Those comparisons showed that the study isolates formed a distinct cluster clearly separated from previously described strains; minimum SNP distance was 20,410 to the closest reference genome (Figure 2).

Genomic comparison with reference strain *D. congolensis* DSM 44180 yielded an average nucleotide identity of 94.55%, below the species delineation threshold of 95%–96% (*9*). Digital DNA–DNA hybridization values among study isolates were 99.9%–100%, whereas values compared with the reference strain were 58.7%, below the 70% threshold for species delineation (*9*). Those findings suggest that the isolates are genetically distinct from currently described *D. congolensis* strains and are consistent with a potentially novel *Dermatophilus* taxon, although further taxonomic characterization would be required to formally define a new species.

## Conclusions

We report human cases of dermatophilosis for which no animal exposure was identified and which are suspected to have been sexually acquired. All cases occurred in MSM with high exposure to STIs, several patients reported partners with similar symptoms, and lesions were commonly located in sites exposed during sexual contact. Those features were similarly described for other considered zoonotic pathogens that emerged as sexually transmissible, such as mpox virus (*10*) and *Trichophyton mentagrophyte*s (*11*). As has already been observed, MSM are particularly vulnerable to such emerging infections, probably because of the complexity of their sexual networks (*12*).

Attendance at sexual venues might have been a factor in transmission in this cluster. In particular, 8 patients developed lesions shortly after visiting a sauna, where humid conditions could favor the release and environmental persistence of infective *Dermatophilus* zoospores (*13*,*14*). Indirect transmission might occur via contaminated surfaces; fomite-mediated outbreaks have been described in animals (*15*). However, based on the anatomic distribution of lesions, direct skin-to-skin contact during sexual activity likely represents the main route of transmission.

All cases in this series were mild and resolved easily without complications. Most patients responded well to short courses of commonly used antibiotics, including β-lactams or doxycycline. That finding is consistent with previous reports of human dermatophilosis as a mostly benign condition (*2*–*6*). Although dermatophilosis might resolve spontaneously over several weeks, accelerated recovery through antibiotic therapy could help reduce transmission within the community. Antimicrobial susceptibility testing showed a broad susceptibility profile to several antibiotic classes, suggesting that standard oral treatments remain effective. In the case of mupirocin, the finding of a high MIC suggests that this topical antibiotic might not be the most suitable treatment option.

Genomic findings support recent *Dermatophilus* transmission among humans. The WGS of isolates showed a close genetic relationship, consistent with a recent common ancestor and short transmission chains. The isolates formed a well-supported phylogenetic cluster distinct from publicly available *D. congolensis* genomes, suggesting the circulation of a single lineage rather than multiple independent introductions and representing a novel taxon within the genus *Dermatophilus*. This previously undescribed taxon circulating in humans could potentially contribute to the epidemiologic pattern observed, although further studies are needed to clarify its ecologic niche, host range, and transmission dynamics.

In summary, this cluster of genetically closely related cases of dermatophilosis within sexual networks suggests that this condition might be emerging as a sexually transmissible infection, although environmental transmission cannot be excluded. Because clinical manifestations can be nonspecific and laboratory identification is uncommon in STI clinics, cases could remain unrecognized. Clinicians should therefore suspect dermatophilosis in MSM who have a folliculitis-like pustular rash involving genital or adjacent areas and should consider oral antibiotic treatment and comprehensive STI screening. Cross-border surveillance could help determine whether similar cases are occurring elsewhere.

Appendix Additional information for suspected sexual transmission of dermatophilosis among men who have sex with men, Barcelona, Spain, 2025–2026.
